# Modeling Obesity-Associated Ovarian Dysfunction in *Drosophila*

**DOI:** 10.3390/nu14245365

**Published:** 2022-12-16

**Authors:** Huanju Liu, Jiajun Li, Xinyue Chang, Feng He, Jun Ma

**Affiliations:** 1 Women’s Hospital and Institute of Genetics, Zhejiang University School of Medicine, Hangzhou 310058, China; 2 Zhejiang Provincial Key Laboratory of Genetic and Developmental Disorder, Hangzhou 310058, China; 3 ZJU-UOE Institute, Zhejiang University School of Medicine, Haining 314400, China; 4 Women’s Reproductive Health Research Laboratory of Zhejiang Province, Hangzhou 310006, China; 5 Zhejiang University-University of Toronto Joint Institute of Genetics and Genome Medicine, Hangzhou 310058, China

**Keywords:** obesity, ovarian dysfunction, insulin resistance, central composite design, *Drosophila*, animal model

## Abstract

We perform quantitative studies to investigate the effect of high-calorie diet on *Drosophila* oogenesis. We use the central composite design (CCD) method to obtain quadratic regression models of body fat and fertility as a function of the concentrations of protein and sucrose, two major macronutrients in *Drosophila* diet, and treatment duration. Our results reveal complex interactions between sucrose and protein in impacting body fat and fertility when they are considered as an integrated physiological response. We verify the utility of our quantitative modeling approach by experimentally confirming the physiological responses—including increased body fat, reduced fertility, and ovarian insulin insensitivity—expected of a treatment condition identified by our modeling method. Under this treatment condition, we uncover a *Drosophila* oogenesis phenotype that exhibits an accumulation of immature oocytes and a halt in the production of mature oocytes, a phenotype that bears resemblance to key aspects of the human condition of polycystic ovary syndrome (PCOS). Our analysis of the dynamic progression of different aspects of diet-induced pathophysiology also suggests an order of the onset timing for obesity, ovarian dysfunction, and insulin resistance. Thus, our study documents the utility of quantitative modeling approaches toward understanding the biology of *Drosophila* female reproduction, in relation to diet-induced obesity and type II diabetes, serving as a potential disease model for human ovarian dysfunction.

## 1. Introduction

Oogenesis is a long and energy-consuming process essential to the propagation of an animal species [1,2,3]. Being a major commitment to the allocation of a significant quantity of bio-resources and a first step toward safeguarding orderly embryonic development for the offspring, oogenesis is a highly regulated, complex process involving the interactions among different tissues [4,5,6]. It is highly sensitive to the availability of nutrients. Either malnutrition or obesity can adversely impact this process and, consequently, female fertility. For example, it has been shown that, in *Drosophila*, starvation or high-sugar diets can each reduce the number of eggs a female produces over her lifespan [7,8]. Female fertility in humans is also known to be sensitive to nutrients and metabolic homeostasis, and metabolic disorders induced by either genetic or life-style factors have been associated with several types of ovarian dysfunction [9,10].

*Drosophila* and humans share similar organs or tissues participating in the regulation of metabolic homeostasis and female reproduction [11,12]. They include the ovary, adipose tissues (fat body in *Drosophila*), *β*-pancreatic islet cells (insulin-producing cells in *Drosophila*), gut, and blood (hemolymph in *Drosophila*). *Drosophila* has been successfully used to model metabolic disorders, and a high-sugar diet can cause insulin resistance [8,13,14,15]. In addition, it is well-documented that oogenesis is dependent on the insulin signaling pathway in *Drosophila* [13,16,17,18,19]. Thus, the relationship between high-sugar diet and egg production defects may be viewed conveniently, though likely incompletely, through insulin resistance (IR) and type II diabetes (T2D). In several human female reproductive conditions, there exists a triangular relationship involving obesity (Ob), T2D, and ovarian dysfunction (OD). These three aspects tend to be intricately associated with one another in a complex, interactive way that compounds the heterogeneous nature of OD syndromes [20,21,22]. One common OD is polycystic ovary syndrome (PCOS), which is characterized by oligo-ovulation/anovulation, a polycystic ovary, and androgen excess [10,23]. Due to the heterogenous nature of this disorder, a clinical diagnosis of PCOS needs to satisfy at least two of these three features, according to the Rotterdam criteria [23]. Meanwhile, around 50–75% of patients with PCOS are obese or have impaired insulin sensitivity [24,25], and obese females are at higher risk for fertility issues [26] and T2D [27]. Women with T2D also tend to have an increased likelihood of anovulation [28], and those with subfertility have an increased risk for T2D [29].

Here, we aim to model the triangular relationship among Ob, T2D, and OD in *Drosophila* through systematic diet manipulations and quantitative modeling of their pathophysiological responses. While the connection between a high-sugar diet and T2D is well-established in *Drosophila* [15,30,31], the contribution of protein—another high-energy component in *Drosophila* diet—to this triangular relationship remains to be fully resolved, particularly with Ob being an indispensable player in this relationship. We design experiments to integrate the use of protein and sugar toward establishing a *Drosophila* model to capture this triangular relationship, referred to as the Ob-T2D-OD model. Through quantitative measurements and data fitting guided by the central composite design (CCD) method, we identify a treatment condition based on the quantitative modeling approach and experimentally verify the three aspects of pathophysiology. We describe an oogenesis phenotype observed under this treatment condition, with an accumulation of immature oocytes and a halt in the production of mature oocytes, a phenotype that resembles the key aspects of PCOS in human patients. Our quantitative measurements also reveal new insights into how the two high-energy food sources—protein and sucrose—interact in impacting the different aspects of the pathophysiology when they are considered as an integrated response. In addition, we provide evidence suggesting a temporal order of onset timing of the three aspects of diet-induced pathophysiology, likely reflective of complex interactions within the triangular relationship. We will discuss the implications of our findings in relation to the understanding of PCOS.

## 2. Materials and Methods

### 2.1. Fly Stocks and Husbandry

The *Drosophila* strain *w^1118^* (BL3605) was used throughout this study. Flies were raised at 25 °C and 60% relative humidity with a 12–12 light/dark cycle. The diet for normal husbandry (ND) contained 5 g/dL corn flour, 1 g/dL agar, 2.45 g/dL dry yeast, 0.725 g/dL white sugar, and 3 g/dL brown sugar. The manipulated diets for CCD experiments contained 8.6 g/dL corn flour, 1.2 g/dL agar, and designed concentrations (see below) of dry yeast and pure sucrose (Sangon Biotech-A502792, Shanghai, China).

### 2.2. Central Composite Design

A CCD design with *k* factors requires a total of *C* + 2*^k^* + 2*k* experiments, where *C* is the number of replicates at the center point of the cubic design space recommended for 5–6; 2*^k^* is the number of factorial points representing the experimental domain; 2*k* is the number of “star” points allowing estimation of curvature, which are axial points outward-extended from the face centers of the cube [32]. The distance from a star point to the center point is 2*^k^*^/4^-fold of the center-to-face distance for each factor. In our design (*k* = 3), the 20 runs included eight factorial points, six start points, and six replicate center points, with two responses (*Bf* and *F*) measured for each run.

In the current study, we evaluated three variables, *S*, *P*, and *D* (see Results and Discussion for details), and our parameter space was chosen based on the following considerations: (1) the range of *S* was set to be as large as possible; (2) excessive *P* was avoided to prevent uncontrolled effects associated with food texture; and (3) a range of *D* was chosen to allow the coverage of the dynamic changes in pathophysiological responses. Accordingly, *S* = 30 ± 17.5 g/dL (center ± center-to-face distance), *P* = 2 ± 1 g/dL, and *D* = 5 ± 2 d were chosen. After this parameter space was augmented by six star points each, with a 1.682-fold dilation, a total of 20 experimental runs were designed (Tables S1 and S2). Design-Expert v12.0.3 was used to randomize the actual layout of experimental runs.

### 2.3. Measurements of Fertility and Body Fat in CCD Experiments

Adults within 1 day of eclosion were collected and free to mate on ND for 3 days. Females were then isolated and distributed into vials (25 × 95 mm), each with ~6 flies on the manipulated diet. Vials were replaced with fresh food of the same formula each day until the time of measurements. Prior to *F* measurement, each vial was replaced with ND food, from which the number of flies alive and the number of eggs produced within a 24-h period were counted. For each experimental run, 4~7 vials were measured as independent replicates.

For *Bf* measurement, flies from each experimental vial were weighed and stored at −80 °C. On the day of measurement, the collected samples were homogenized in iced PBS and then centrifuged at 4 °C. From each sample, three aliquots of 5 μL supernatants were subjected to triglycerides assay, according to the manufacturer’s instructions (XinFan-XFC121, Shanghai, China). *Bf* was calculated by $\frac{C_{\mathrm{TG}}\;\left(nmol\cdot \mu L^{-1}\right)\times \;V\;\left(\mu L\right)}{\mathrm{body}\;\mathrm{weight}\;\mathrm{mg}}$, where C_TG_ is the TG concentration and V is the added PBS volume.

### 2.4. Statistical Modeling of CCD Data

Design-Expert was used to perform statistical analyses. Externally studentized residuals (ESR) were used to detect unexpected trends or outlying observations. A series of diagnostics plots, including ESR plot (Figure S1A,B) and normal probability plot (Figure S1C,D), were generated. According to the instructions of Design-Expert, runs #2 and #13 for *Bf* and runs #17 and #19 for *F* were excluded from modeling; no further transformation was made.

For each response, *Bf* or *F*, a quadratic model was suggested over linear, two-factor-interaction or cubic model by Design-Expert. ANOVA, adjusted *R*^2^ (indicating amount of variation explained by the model), predicted *R*^2^ (indicating amount of variation in predicted data explained by the model), and adequate precision (requiring a value greater than 4 to indicate a high signal-to-noise ratio) all demonstrated good fitting of each model (Table S3). Independent ANOVA was also performed against each term of the model. The results suggested that all terms were significant in one or both of the models. Therefore, no term reduction or further optimization was considered.

The resulting models were expressed using either coefficients with actual units or coefficients with one single unit that was coded for all three variables as $\mathrm{coded}=\frac{\mathrm{actual}\;\mathrm{setting}\;-\;\mathrm{average}\;\mathrm{actual}\;\mathrm{setting}}{\left(\mathrm{range}\;\mathrm{between}\;\mathrm{low}\;\mathrm{and}\;\mathrm{high}\;\mathrm{actual}\;\mathrm{settings}\right)/2}$. The actual models (Equations (1) and (2)) were used to identify optimal conditions from the integration of the two responses.

(1) $$y_{Bf}=47.0318+0.4507x_{1}-12.1659x_{2}-3.2591x_{3}+0.5661x_{1}x_{2}-0.06036x_{1}x_{3}-1.0766x_{2}x_{3}-\;0.0162x_{1}{}^{2}+0.4255x_{2}{}^{2}+0.7499x_{3}{}^{2}$$

(2) $$y_{F}=7.01887-0.315118x_{1}+3.50601x_{2}-0.342871x_{3}-0.088894x_{1}x_{2}+0.015099x_{1}x_{3}-0.454678x_{2}x_{3}\;+0.004912x_{1}{}^{2}+0.985154x_{2}{}^{2}-0.033222x_{3}{}^{2}$$

In the coded models (Equations (3) and (4)), the coded coefficients represent the relative contributions of the corresponding terms. The interaction between *S* and *P* (*x*_1_*x*_2_) contributed the most to *Bf* among all variable terms, but contributed less and in an opposite direction to *F*.

(3) $$y_{Bf}=39.97+5.44x_{1}+1.14x_{2}+0.5505x_{3}+9.91x_{1}x_{2}-2.11x_{1}x_{3}-2.15x_{2}x_{3}-4.9\;5x_{1}{}^{2}+0.4255x_{2}{}^{2}\;+3.00x_{3}{}^{2}$$

(4) $$y_{F}=2.78-2.15x_{1}+2.51x_{2}-2.26x_{3}-1.56x_{1}x_{2}+0.5285x_{1}x_{3}-0.9094x_{2}x_{3}+1.50x_{1}{}^{2}+0.9852x_{2}{}^{2}\;-0.1329x_{3}{}^{2}$$

Four desirability functions were used to integrate the two responses of *Bf* and *F*: $d\left(Bf_{high}\right)=\frac{Bf-min\left(Bf\right)}{\max\left(Bf\right)-min\left(Bf\right)}$, $d\left(Bf_{low}\right)=\frac{max\left(Bf\right)-Bf}{\max\left(Bf\right)-min\left(Bf\right)}$, $d\left(F_{high}\right)=\frac{F-min\left(F\right)}{\max\left(F\right)-min\left(F\right)}$, and $d\left(F_{low}\right)=\frac{max\left(F\right)-F}{\max\left(F\right)-min\left(F\right)}$.

### 2.5. Measurement of Continued Egg Production

Under *S*_2_*P*_3_ and *S*_35_*P*_3_, we measured continued egg production from the same females on a daily basis for a 6-day period. The procedure was identical to that used in CCD experiments, except that egg laying was carried out on the manipulated diet, instead of ND. Thus, in this continued measurement, *D* = *n* refers to the number of eggs during the 1-day period of (*n* − 1 to *n*) on the specified diet, whereas in *F* measurement it refers to the number of eggs during the 1-day period of (*n* to *n* + 1) on ND.

### 2.6. Staining and Quantification of Lipid Droplets in the Fat Body

Fat body tissues were freshly dissected from females in the four selected experimental runs: *S*_2_*P*_3_*D*_1_, *S*_2_*P*_3_*D*_6_, *S*_35_*P*_3_*D*_1_, and *S*_35_*P*_3_*D*_6_. Dissected tissues were immediately fixed in PBS with 4% formaldehyde for 20 min at room temperature (RT) and washed in PBST for 10 min × 3. Then, the samples were stained with 1 μg/mL Nile red (Sigma-72485, St. Louis, MO, USA) for 30 min. After another three washes, the samples were incubated with phalloidin (BIORIGIN-BN1053, Beijing, China, 1:200 in PBT) for 1 h. After 3 washes with PBST, the samples were mounted in DAPI-containing medium (Beyotime-PO131, Shanghai, China). Images were acquired with a magnification of 400 under the confocal microscope (Olympus-FV1000, Tokyo, Japan). The sizes of LDs were quantified by ImageJ v1.53.

### 2.7. Measurement of Food and Energy Intake

We measured feeding under four conditions *S*_2_*P*_3_*D*_1_, *S*_35_*P*_3_*D*_1_, *S*_2_*P*_3_*D*_6_, and *S*_35_*P*_3_*D*_6_, as follows. Females completed their specified treatment with the last 24 h on the food containing 0.5 g/dL Brilliant Blue (Solarbo-E8500, Beijing, China). The collected 5–6 flies in each vial were homogenized in PBS on ice and then centrifuged at 4 °C. From the supernatants, optical density at 630 nm (OD630) was measured. Averaged food intake per fly per day was determined from the linear ranges of pre-established standard curves (food concentration in PBS vs. OD630) for *S*_2_*P*_3_ and *S*_35_*P*_3_, respectively. Caloric intake was calculated using the *Drosophila* dietary composition calculator [33].

### 2.8. Hemolymph Glucose Measurement

Liquid nitrogen freezing, followed by vortex, was used separate the head from body of 5–6 weighted females in an Eppendorf tube. PBS solution (10 μL/female) was added, followed by centrifugation at 3000× *g* for 6min (4 °C). The supernatant was heated at 80 °C for 7 min and centrifuged at 13,000× *g* for 15 min. Glucose detection of the supernatant was based on GOD-POD methods (LEAGENE TC0711, Beijing, China), and sample absorbance under 510 nm was measured with the microplate reader (UMR-9600, Hangzhou, China). Hemolymph glucose content was calculated through the reference standard and normalized to body weight (BW).

### 2.9. Ovary Morphology Imaging and Ovarian Stage Identification

Ovaries were dissected within PBS under stereomicroscope (Nikon C-PSN, Tokyo, Japan) and imaged under 40× microscope (Nikon SMZ18, Tokyo, Japan) with the camera (Nikon DS-Ri2, Tokyo, Japan). Ovarioles were separated from whole ovaries within DAPI-containing medium on slides. Egg chambers of whole ovaries were imaged with microscopes (ZESS Axio-Observer, Olympus-FV1000) to obtain both DAPI and bright field. Egg chamber size was measured by Image J, and it was used as a feature in stage identification [34]. Stages 1–7 were identified by their overall size, in combination with manual verification; stages 8 and 9 were identified according to the presence or absence of the nuclei surrounding the anterior nurse cells and stages 10–14 were identified based on morphology.

### 2.10. Insulin Sensitivity Assay of Dissected Ovaries

Ovaries from females cultured on *S*_2_*P*_3_ and *S*_35_*P*_3_ diets from *D* = 0 to *D* = 6 were freshly dissected in Schneider’s *Drosophila* medium (Gibco, USA) with serum supplement (GEMINI−900–108), and then incubated with or without 0.5 μM recombinant human insulin (NJDULY-P0029, Nanjing, China) for 15 min at RT. After three washes in PBS, the tissue samples were lysed in RIPA buffer (FDBIO-FD009, Hangzhou, China) with phenylmethanesulfonyl fluoride (FDBIO-FD0100), protease inhibitor mix (FDBIO-FD1001), and protein phosphatase inhibitor mix (FDBIO-FD1002) on ice. After centrifugation at 13,000× *g* at 4 °C for 10 min, the supernatants were mixed with loading buffer (FDBIO-FD006) at 98 °C for 5 min. Proteins were separated on 10% SDS-PAGE (FDBIO-FD346) and then transferred to the PVDF membrane (Millipore-IPVH00010, USA). Following blocking in TBST with 5% non-fat milk at RT for 1.5 h, the membrane was added with the primary antibody and incubated overnight with rocking at 4 °C. After three washes in TBST, the membrane was incubated with anti-mouse or anti-rabbit HRP-conjugated secondary antibodies (GenScript-A00160 and A00098, respectively) at RT for 1 h. The membrane was then washed three times in TBST and visualized using ECL Western Blotting Substrate (Vazyme-E411, Nanjing, China). Blots were scanned using a gel imaging system (QinXiang-ChemiScope6000, Shanghai, China). Primary antibodies used in this study were: phospho-AKT (Ser473) (Cell Signaling Technology-#9271, MA, USA), total Akt (Cell Signaling Technology-#4691), and α-tubulin (Beyotime-AT819).

### 2.11. mRNA-seq Library Preparation, Sequencing and Analysis

Each total RNA sample was extracted from freshly dissected ovaries of ~20 females under the selected condition with RNAiso Plus (Takara, Tokyo, Japan). mRNA-seq libraries were prepared with VAHTS V3 Library Prep Kit (Vazyme, Nanjing, China). Sequencing was performed with PE150 chemistry by NovaSeq6000 (Illumina, San Diego, CA, USA). The clean reads were trimmed for adaptors and filtered with fastp v0.20.1 [35] and then aligned to the dmel_r6.34 reference genome with HISAT2 v2.2.1 with default parameters [36]. The counts of reads mapped to protein-coding genes were summarized with featureCounts v2.0.1 [37]. Differential gene expression (DEG) analysis was performed with DESeq2 v1.32.0 [38]. Gene Ontology and KEGG enrichment analyses of differentially expressed genes (|log_2_FoldChange| > 0.5 and *p*-value < 0.05) were performed with the R package clusterProfiler v4.2.2 [39]. Top GO or KEGG terms with the smallest Benjamini-Hochberg-adjusted *p*-values were graphically displayed. Complete information of DEG, GO, and KEGG analyses are presented in Supplementary Table S1. 

### 2.12. Other Statistical Information and Visualization Tools

Quantified results were reported as mean ± standard error of the mean (SEM), unless specified otherwise. Two-tailed *t*-test and two-way ANOVA were used in the analysis. Bar plots were generated with GraphPad 9. Stacked bar charts and line plots with confidence interval were generated with Origin Lab Pro. Diagrams were generated with Adobe Illustrator and, when necessary, aided by BioRender “https://app.biorender.com/biorender-templates (accessed on 8 March 2022)” for illustrative artwork.

## 3. Results and Discussion

### 3.1. CCD Analysis Evaluating the Role of Sucrose and Protein on Body Fat and Fertility

We utilized the CCD method to quantitatively evaluate the effect of sucrose and protein (yeast) on body fat and female fertility. CCD is a response surface methodology frequently used to evaluate multiple variables impacting an outcome to facilitate optimizations [40]. Here, we used three independent variables: sucrose concentration in the food (denoted as *S*), yeast concentration representing protein content (denoted as *P*), and treatment duration on a given diet (denoted as *D*). At each treatment condition with a set of predetermined values for *S*, *P*, and *D*, we measured the body fat (denoted as *Bf*), which is the amount of triglyceride per unit of total body weight, and fertility (denoted as *F*), which is the average number of eggs produced per female per day. Since the effect of a high-sucrose diet on T2D is well-documented in *Drosophila* [31,41], we did not perform direct measurements of this aspect during CCD analysis (see below for experimental verification).

With three independent variables, we employed 20 treatment conditions (runs), specified under the CCD methodological framework (Table S1). Each run performed no fewer than three replicates or independent measurements, the mean of which is referred to as a datapoint, as shown in Table S2. As detailed in Materials and Methods, flies within one-day emergence were transferred to bottles containing normal diet (ND). Three days later, 5~7 females were selected and placed in individual vials, each constituting a run, as designed by CCD. Upon the completion of a treatment, the females were then placed back on ND for one day to obtain the average number of eggs per female during this one-day period as the *F* measurement or taken immediately for *Bf* measurements upon freezing. The experimental design and the corresponding results, which were derived from a total of 158 separate measurements, are summarized in Table S2.

After verification of our experimental measurements (Figure S1A–D; see Materials and Methods for further details), the associations between the three independent variables and the two responses were modeled with a quadratic function in Equation (5),

(5) $$y=\sum_{i=1}^{k}\beta_{i}x_{i}+\sum_{i=1}^{k}\beta_{ii}x_{i}{}^{2}+\sum_{i<j}^{k}\beta_{ij}x_{i}x_{j}+\beta_{0}$$

where y is *Bf* or *F*; *x_i_* is *S*, *P*, or *D*; $\beta_{i}\;,\beta_{ii}$, and $\beta_{ij}$ are the coefficients for $x_{i}$ (linear), $x_{i}{}^{2}$ (quadratic), and $x_{i}x_{j}$ (first-order interaction), respectively; $\beta_{0}$ is a model constant. The values of these coefficients (Equations (1)–(4)) and the detailed modeling strategy are summarized in Materials and Methods. The resulting models, as well as each term individually, were tested for statistical fitness with ANOVA (Table S3). F-tests revealed a *p*-value of < 0.001 in each response, suggesting that the quadratic fitting was statistically significant in each case. Scatter plots of the predicted values against experimental measurements exhibited an excellent linearity (*R*^2^ = 0.94 and 0.99 for *Bf* and *F*, respectively; Figure S1E,F).

### 3.2. Evaluating Body Fat and Fertility as Distinct Responses or As an Integrated Response under Different Scenarios

**Figure 1:**
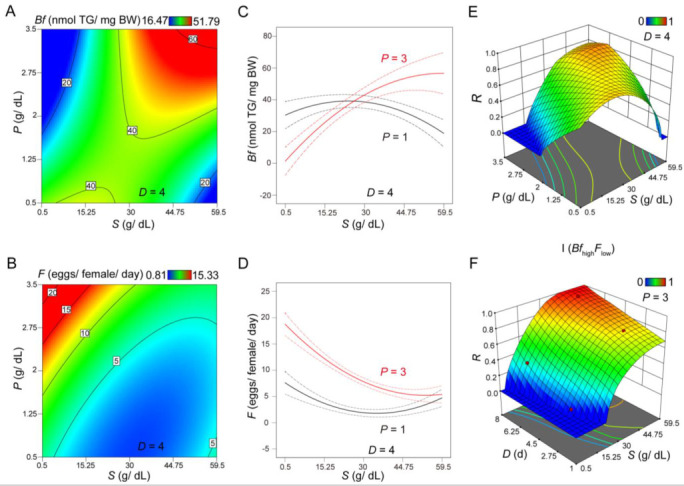
Characterization of *Bf* and *F* under various feeding treatment. (**A**,**B**) Contour plots of *Bf* (**A**) or *F* (**B**) at *D* = 4. The *X*-axis represents *S*, *Y*-axis represents *P*, and the color represents levels of *Bf* (**A**) or *F* (**B**). (**C**,**D**) Interaction plots of *S* and *P* on *Bf* (**C**) or *F* (**D**) at *D* = 4. The *X*-axis represents *S; Y-axis* represents *Bf* (**C**) or *F* (**D**). The two lines show the response of *Bf* (**C**) or *F* (**D**) to *S* at different levels of *P*. Dashed lines represent 95% confidence intervals. (**E**,**F**) Integrated 3D surface plots under Scenario I, showing *R* as a function of *S*-*P* (**E**) or *S*-*D* (**F**), respectively. Here, a high value of *R* represents a high desirability for achieving *Bf*_high_*F*_low_. *D* = 4 in panel **E** and *P* = 3 in panel **F**.

Graphic presentations of the two responses for 4-day treatment (*D* = 4) are shown in Figure 1A,B). These results revealed that, contrary to what would have been expected in a simplistic manner [8,13,42,43], an increase in *S* did not necessarily cause a significant increase in *Bf* (Figure 1A) or reduction in *F* (Figure 1B). Interaction plots for *Bf* (Figure 1C) and *F* (Figure 1D) further supported the suggestion that the effects of *S* on these two physiological responses depended on *P*. The interaction between *P* and *S* in impacting *Bf* or *F*, documented here (also see below) and elsewhere [44], supported a need for a systems approach, as described in the current study.

To evaluate how the three variables (*S*, *P*, and *D*) interact in driving *Bf* and *F* as an integrated response toward an optimal outcome, we performed an integration of the multiple responses, as in Equation (6),

(6) $$R=\sqrt[{n}]{\prod_{i}^{n}d_{i}\left(y_{i}\right)}$$

where *y_i_* denotes each response and *d_i_* is the corresponding desirability function that transforms *y_i_* to a scaled value between 0 and 1. Here, *d*_i_(*y*_i_) = 1 always represents the most desirable, but the exact function for each given response is also specific to a given desire. For two responses, *Bf* and *F*, there are four possible scenarios, each with a distinct desired outcome: I (*Bf*_high_*F*_low_), II (*Bf*_low_*F*_high_), III (*Bf*_high_*F*_high_), and IV (*Bf*_low_*F*_low_) (see Materials and Methods for their desirability functions). Among the four possible scenarios, Scenario I had two of the features in our desired Ob-T2D-OD model (see below for T2D verification).

Given the two responses that we determined as functions of *S, P*, and *D* (Equations (5) and (6)), a response surface methodology was used to visualize and identify optimized values of *S, P*, and *D* under different scenarios (when the respective *R* approaches or equals 1). Figure 1E,F display *R* on the *S-P* and *S-D* planes, respectively. The *S*-*P* plot (Figure 1E) shows that *P* = 3 approaches an optimized condition for obtaining a desired effect of high *S* toward simultaneously achieving *Bf*_high_ and *F*_low_. The *S-D* plot (Figure 1F) shows that, at *P* = 3, the higher *S* and longer *D*, the higher the desirability value *R*. Importantly, our results showed that *R* no longer responded significantly to *S* when *S* > 35. Together, these results led to the selection of the treatment condition *S*_35_*P*_3_*D*_6_ (denoting *S* = 35, *P* = 3 and *D* = 6) to represent our Ob-T2D-OD model, and the experimental validations were performed for individual aspects of the pathophysiology, as described further below.

**Figure 2:**
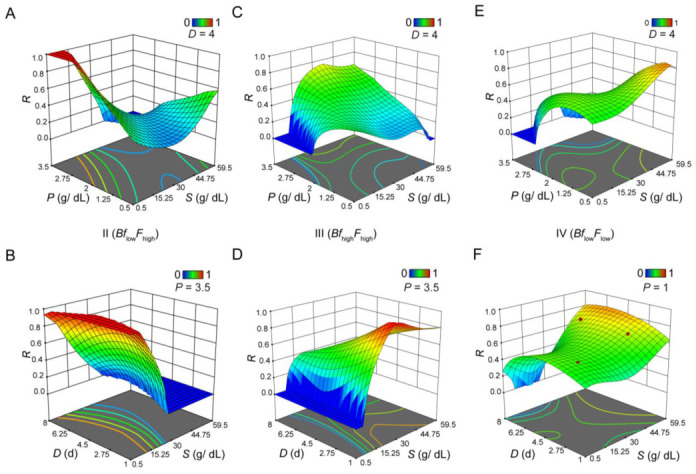
Integration analysis of *Bf* and *F* with different phenotype preference. Shown are integrated 3D surface plots showing *R* as a function of *S*-*P* (**A**,**C**,**E**) or *S*-*D* (**B**,**D**,**F**) under three different scenarios (**A**–**B** for Scenario II, **C**–**D** for Scenario III, and **D**–**F** for Scenario IV). A high *R* value indicates a high desirability for the given scenario. For each panel, values for *D* or *P* are indicated.

The other three scenarios provided additional opportunities for us to evaluate the complex interaction between *S* and *P* in their physiological impact (Figure 2). In both Scenarios II and III, a high *P* (*P* = 3.5) was necessary for their respective desirable outcomes (Figure 2A,C). In Scenario II, *R* was higher at low *S* (*S* = 1) and did not respond significantly to *D* (Figure 2B). However, in Scenario III, *R* approached its maximal level at approximately *S* = 30 with a small *D* (*D* = 1) (Figure 2D). In this case, an increase in *D* led to a reduction in *R*. To achieve a high value of *R* in Scenario IV (Figure 2E,F), a low *P* (*P* = 1), a high *S* (*S* = 60), and a long *D* (*D* = 6) were needed. These results showed that, within our measurement ranges, *S*, *P*, and *D* were interdependent parameters when driving toward specific outcomes, such as integrated physiological responses. They provided a system view on diet manipulation, impacting multiple aspects of the induced pathophysiology in a quantifiable manner in *Drosophila*.

### 3.3. Experimental Verification of the Ob Aspect of Pathophysiology in the Ob-T2D-OD Model

To evaluate the utility of our quantitative modeling-based approach, we performed experimental verification under our modeling-identified treatment condition *S*_35_*P*_3_*D*_6_, along with its normal-sugar control *S*_2_*P*_3_*D*_6_. We also included their *D* = 1 counterparts as additional tests to facilitate an evaluation of the dynamic progression of the pathophysiological responses. Under our quantitative modeling framework, *S*_35_*P*_3_*D*_6_ was expected to exhibit both obesity and ovarian dysfunction (see below for T2D). To test this directly, we measured properties indicative of pathophysiological responses under each of the four conditions, *S*_2_*P*_3_*D*_1_, *S*_2_*P*_3_*D*_6_, *S*_35_*P*_3_*D*_1_, and *S*_35_*P*_3_*D*_6_.

**Figure 3:**
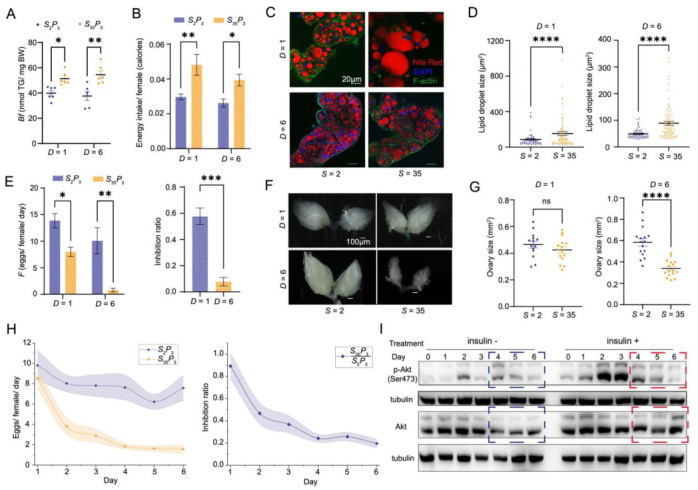
Phenotype verification under the selected treatment conditions. (**A**) *Bf* measurement under the four indicated conditions (ANOVA), * *p* < 0.05, ** *p* < 0.01. (**B**) Calorie intake calculation from feeding quantification with the dye assay under four indicated conditions (ANOVA), * *p* < 0.05, ** *p* < 0.01. (**C**) Nile red staining of lipid droplets in the fat body under *S*_2_*P*_3_*D*_1_, *S*_35_*P*_3_*D*_1_, *S*_2_*P*_3_*D*_6_, *S*_35_*P*_3_*D*_6_. F-actin stained with phalloidin was in green, nucleus stained with DAPI was in blue. Scale bars: 20 μm. (**D**) Measurements and statistical analysis of lipid droplet size under the four indicated conditions (*t*-test), **** *p* < 10^−4^. (**E**) *F* measurement under four conditions (ANOVA). The inhibition ratio of high sucrose on *F* was calculated by the number of eggs produced under *S*_35_*P*_3_ divided by that under *S*_2_*P*_3_ (*t*-test), * *p* < 0.05, ** *p* < 0.01, *** *p* < 10^−3^. (**F**) Comparison of whole ovarian morphology under four conditions. Scale bars: 100 μm. (**G**) Measurement and statistical analysis of ovarian size under four conditions (*t*-test), **** *p* < 10^−4^. (**H**) Continued egg production from day 1 to day 6 on the indicated diet. The inhibition ratio as a function of treatment time under high sucrose diet. See Materials and Methods for further details. (**I**) Shown are Western blotting results detecting p-Akt, Akt, and tubulin of the ovary (*D* = 0 to *D* = 6), with or without the addition of human insulin to the dissected ovaries. Insulin sensitivity was calculated as the ratio of detected protein levels, p-Akt/Akt. Protein levels were normalized to that of tubulin.

Our measurements of whole-body triglyceride (TG) revealed a significant increase under the condition of *S*_35_*P*_3_*D*_6_, relative to *S*_2_*P*_3_*D*_6_ (37.61 ± 3.45 and 54.45 ± 3.04, respectively, adjusted *p*-value = 0.0017; Figure 3A), confirming our model prediction of the obesity phenotype. Our results also revealed that such an increase became detectable, even at *D* = 1 (39.80 ± 2.12 and 51.31 ± 2.13, respectively; adjusted *p*-value = 0.0355; Figure 3A), suggesting an early onset of this phenotype. In *Drosophila*, TG is synthesized from dietary carbohydrates, fatty acids, and proteins, and is stored as intracellular lipid droplets (LDs) in the fat body [45]. To further evaluate our model prediction, we used Nile Red and phalloidin staining to detect LDs in the fat body (Figure 3C). Our results showed an increase in the overall size of LDs at *S*_35_*P*_3_, relative to their *S*_2_*P*_3_ counterparts at both *D* = 1 and *D* = 6 (measured LD sizes were 86.53 ± 11.33 μm^2^ and 153.9 ± 21.72 μm^2^ for *S*_2_*P*_3_*D*_1_ and *S*_35_*P*_3_*D*_1_, respectively; *p*-value < 10^−4^; 49.41 ± 2.446 μm^2^ and 89.18 ± 7.528 μm^2^ for *S*_2_*P*_3_*D*_6_ and *S*_35_*P*_3_*D*_6_, respectively; *p*-value < 10^−4^; Figure 3D). These results further supported our model prediction of the obesity phenotype and an early onset of this aspect of pathophysiology.

We evaluated the feeding property (see details in Materials and Methods) and found that, while *Drosophila* had a reduced food intake at *S*_35_*P*_3_, relative to *S*_2_*P*_3_ (0.075 ± 0.004 mg and 0.041 ± 0.005 mg per female for *S*_2_*P*_3_*D*_1_ and *S*_35_*P*_3_*D*_1_, respectively; adjusted *p*-value = 0.0055; 0.066 ± 0.006 mg and 0.034 ± 0.003 mg per female for *S*_2_*P*_3_*D*_6_ and *S*_35_*P*_3_*D*_6_, respectively; adjusted *p*-value = 0.0302), the total caloric intake per female at *S*_35_*P*_3_ was significantly higher than at *S*_2_*P*_3_, irrespective of *D* (0.030 ± 0.002 Cal and 0.048 ± 0.006 Cal for *S*_2_*P*_3_*D*_1_ and *S*_35_*P*_3_*D*_1_, respectively; adjusted *p*-value = 5.5 × 10^−3^; 0.026 ± 0.002 Cal and 0.039 ± 0.003 Cal for *S*_2_*P*_3_*D*_6_ and *S*_35_*P*_3_*D*_6_, respectively; adjusted *p*-value = 0.0302; Figure 3B). The increased caloric intake was in full agreement with the observed diet-induced obesity.

### 3.4. Evaluation of the OD Aspect of Pathophysiology in the Ob-T2D-OD Model

To evaluate the OD aspect of our model prediction, we measured egg production and ovarian size under the conditions of *S*_2_*P*_3_*D*_6_ and *S*_35_*P*_3_*D*_6_. Again, we included the *D* = 1 counterparts in our experiments. Despite the obesity phenotype at *D* = 1, as shown in Figure 3A,C, we observed only a modest reduction in the number of eggs produced by females at *D* = 1 (*F* = 13.90 ± 1.39 and 8.09 ± 0.88 for *S*_2_*P*_3_*D*_1_ and *S*_35_*P*_3_*D*_1_, respectively; adjusted *p*-value = 0.0314; Figure 3E). In addition, the overall ovarian size was not significantly different between *S*_35_*P*_3_*D*_1_ and *S*_2_*P*_3_*D*_1_ *(*0.425 ± 0.024 mm^2^ and 0.465 ± 0.025 mm^2^, respectively; *p*-value = 0.2644; Figure 3F,G). These results indicated that the onset of obesity was an early event, and at early treatment times, it was not yet sufficient to fully impact female fertility. By comparison, *S*_35_*P*_3_ led to a dramatic reduction in ovary size at *D* = *6* (0.584 ± 0.034 mm^2^ and 0.339 ± 0.019 mm^2^ for *S*_2_*P*_3_*D*_6_ and *S*_35_*P*_3_*D*_6_, respectively; *p*-value < 10^−4^; Figure 3F,G). At *D* = 6, which corresponded to our modeling-selected condition of *S*_35_*P*_3_*D*_6_, the average number of eggs produced per female per day dropped precipitously to no more than one, representing a nearly complete halt *(*10.08 ± 2.47 and 0.79 ± 0.33 for *S*_2_*P*_3_*D*_6_ and *S*_35_*P*_3_*D*_6_, respectively; adjusted *p*-value = 0.0016; Figure 3E*)*. These results directly supported the suitability of our modeling-selected treatment condition in capturing the desired phenotypes; moreover, the severity of the defects in egg production was among the strongest in the available studies [8,13].

### 3.5. Verification of the T2D Aspect of Pathophysiology in the Ob-T2D-OD Model

To verify that *S*_35_*P*_3_*D*_6_ also captured the T2D aspect of the Ob-T2D-OD triangular relationship, we directly measured insulin sensitivity. We dissected ovaries from females, both prior to feeding on *S*_35_*P*_3_ (Day 0) and after being placed on this food for 1 through 6 days. These ovaries were then analyzed for their response to exogenously added insulin through Western blotting analysis, which determined the levels of Akt and its active form p-Akt. The changes in the p-Akt/Akt ratio induced by insulin provided a readout of insulin sensitivity (Figure 3I), as previously described [13]. In our experiments, *S*_35_*P*_3_*D*_6_ resulted in an inability of the ovary to respond to insulin or insulin resistance (IR, indicative of T2D), but this effect did not take place until *D* = 4 (Figure 3I). Importantly, our temporal analysis of insulin sensitivity in the ovary (Figure 3I) suggested that *D* = 4 represented the onset of the T2D aspect of pathophysiology. We further verified T2D in our Ob-T2D-OD model by documenting an increased hemolymph glucose level (2.65 ± 0.22 μg/mg and 3.51 ± 0.08 μg/mg in control (Con, *S*_2_*P*_3_*D*_6_) and Ob-T2D-OD (*S*_35_*P*_3_*D*_6_) females, respectively; *p*-value = 0.0021).

### 3.6. Dynamic Progression of Diet-Induced Pathophysiology in Ob-T2D-OD Model

To obtain a better understanding of the triangular relationship, with respect to the dynamic progression of diet-induced pathophysiology, we measured egg production under *S*_2_*P*_3_ and *S*_35_*P*_3_ conditions in a continuous manner (Table S4 and see Materials and Methods for details). In this analysis, females were kept on the specified food for egg laying without the step of switching to normal food, as performed in the CCD analysis. Our results showed that egg production became significantly affected at *D* = 2 with its maximal effect at *D* = 6, as judged by the inhibition ratio in egg production (*S*_35_*P*_3_/*S*_2_*P*_3_) (Figure 3H). These findings, together with those shown in Figure 3D,I, suggested a temporal order for the onset of the three aspects of pathophysiology in our *Drosophila* disease model, as follows: Ob, initial OD, T2D (IR), and severe OD. Such a temporal order further suggested that IR of the ovary was unlikely the primary or sole cause for ovarian dysfunction in our system, but it may contribute to its further deterioration. Instead, Ob had the earliest onset, which accompanied or slightly preceded a significant reduction in the ovary size and number of eggs produced by the female.

### 3.7. Oogenesis Defect in Ob-T2D-OD Model Resembles That of the Human Condition PCOS

**Figure 4:**
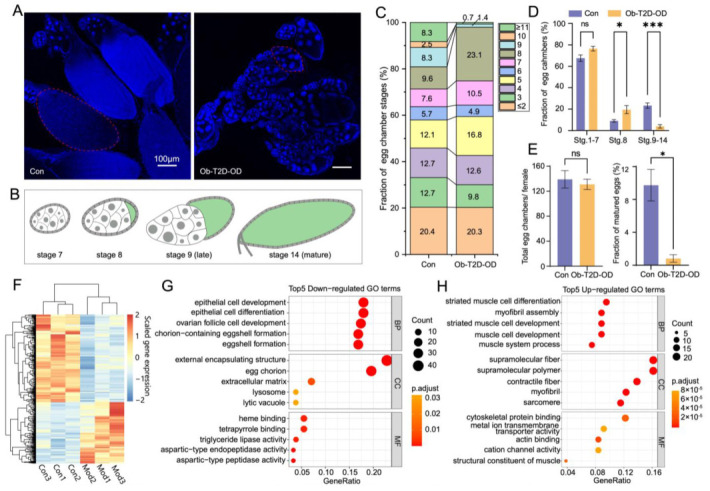
Halted oogenesis and molecular defects in the Ob-T2D-OD *Drosophila* model. (**A**) Confocal microscopy imaging visualizing egg chambers from control (Con) and Ob-T2D-OD females. Imaging under 405 nm and 100× with scale bars: 100 μm. The circled areas provide examples of egg chambers at stage 14 (Con) or stage 8 (Ob-T2D-OD). (**B**) Schematic illustration of egg chambers at the stages shown. Morphological difference is readily recognizable based on egg chamber size and the presence of follicle cell nuclei surrounding the anterior nurse cells. (**C**) Percentages of egg chambers at different stages from the ovaries of a representative control or Ob-T2D-OD female with a stacked bar chart. (**D**) Statistical analysis of stage comparations in the Con and Ob-T2D-OD (ANOVA), * *p* < 0.05, *** *p* < 10^−3^. (**E**) Statistical analysis of total egg chamber numbers and fractions of stage 14 in the Con and Ob-T2D-OD females (*t*-test), * *p* < 0.05. (**F**) Hierarchical clustering of genes that were significantly differentially expressed (|log2FoldChange| > 0.5 and *p*-value < 0.05) in ovaries between the control group (*S*_2_*P*_3_*D*_6_) and the Ob-T2D-OD model group (*S*_35_*P*_3_*D*_6_). Each group consists of three independent replicates. (**G**,**H**) GO enrichment analysis of genes that were significantly down-regulated (*n* = 253) and up-regulated (*n* = 177) under *S*_35_*P*_3_*D*_6_. Shown are the top 5 terms with the smallest Benjamini-Hochberg adjusted *p*-values under each category of Biological Process (BP), Cellular Component (CC), and Molecular Function (MF).

The *Drosophila* ovary consists of ~16 ovarioles, each containing a string of developing egg chambers at distinct stages with morphologically recognizable features [34,46] (see Materials and Methods for details). *Drosophila* oogenesis can be divided into 14 stages (Figure 4B), and these developing egg chambers resemble the growth of human follicles [47]. To gain insights into the defect in egg production under the treatment of *S_35_P_3_D_6_* from a developmental perspective, we quantified the distribution of oogenesis stages in the dissected ovaries (Figure 4A). We measured individual egg chamber’s overall size and characterized their nuclear morphology for stage identification, as before [34]. Figure 4C shows the stage distributions of egg chambers from Con and Ob-T2D-OD females.

Our results revealed a significant increase in the fraction of egg chambers at stage 8, from 9.04 ± 1.15% in the control group to 19.54 ± 3.78% (adjusted *p*-value = 0.036; Figure 4D). As a consequence, the fraction of egg chambers at stages 9–14 was severely reduced, from 23.36 ± 2.47% to 3.94 ± 1.64% (adjusted *p*-value = 4 × 10^−4^; Figure 4D). Mature oocytes became virtually undetectable in the Ob-T2D-OD group, exhibiting a drop from 9.74 ± 1.92% in the control group to less than 1% (*p*-value = 0.011; Figure 4E). Despite a reduction in the number of matured egg chambers in the Ob-T2D-OD group, the total number of egg chambers remained unchanged (139 ± 14 and 131 ± 8 for Con and Ob-T2D-OD, respectively; *p*-value = 0.649; Figure 4E). These results documented a special phenotype of the Ob-T2D-OD females, as characterized by both a stalled oogenesis at stage 8 and an accumulation of immature oocytes at this stage (Figure 4C,D).

Stage 8 of oogenesis marks the onset of significant oocyte growth in its overall size (Figure 4B) [48]. It has been reported that stage 8/9 acts as a nutrient checkpoint, at which an egg chamber can proceed further development or undergo apoptosis upon starvation [49]. While both starvation and our Ob-T2D-OD disease model involve stage 8 in leading to a defect in mature egg production [50], our observations pointed to important differences. In our disease model, there was a significant accumulation of egg chambers at stage 8, with a minimal effect on egg chambers at stages 1–7 (Figure 4D). By contrast, it is well-documented that, under starvation conditions, egg chambers at stages 8 and 9 undergo apoptosis, accompanied by a heightened level of autophagy [49,51]. To gain a better understanding of the molecular defects in our disease model, we performed an RNA-seq analysis in the ovaries of control and disease model females. Figure 4F shows a heatmap exhibiting clusters of differentially expressed genes, with the results of the GO enrichment analysis shown in Figure 4G,H (see Table S5 for a complete list of these genes and their GO and KEGG analyses). These results showed a significant transcriptional dysregulation in our Ob-T2D-OD model. Importantly, unlike what would be expected of starvation, genes associated with apoptosis, autophagy, or lysosome were not on the up-regulated list. In fact, autophagy- (e.g., Atg18b) and lysosome-related (e.g., Tsp42Ea) were down-regulated in our data (Table S5). These results further supported the notion that the oogenesis phenotype in our disease model was distinct from that caused by starvation, suggesting that, in molecular terms, the nutrient checkpoint of stage 8 was impacted distinctly by starvation and high-caloric diet, leading to a failure to produce mature eggs.

### 3.8. Implications of the Ob-T2D-OD Disease Model in Relation to PCOS and Previous Studies

In both humans and *Drosophila*, somatic cells derived from stem cells proliferate to form an epithelial monolayer that supports oocyte development and maturation [52,53]. During ovulation, the mature oocyte is released from the egg chamber or ovarian follicle, and the remaining cells become corpus luteum. There is a delicate interaction between the developing oocyte and these surrounding somatic cells in responding to nutritional and metabolic inputs [54,55,56]. These somatic cells in *Drosophila* are called follicle cells, while those in humans include granulosa and theca cells [57,58]. The oogenesis phenotype in our Ob-T2D-OD model included both an accumulation of immature oocytes and a lack of mature oocytes. These two aspects bear resemblance to the human condition PCOS, particularly with regard to the clinical criteria of oligo-anovulatory infertility and polycystic ovarian morphology [59]. Unlike human PCOS, where the overall ovary size is enlarged, resulting from the accumulated, immature oocytes, the *Drosophila* ovaries are actually smaller in our case. This difference is purely reflective of a nearly complete lack of mature (or late stage) oocytes that, under normal conditions, would take up the bulk of the volume of the ovary in *Drosophila* [60,61,62]. In this context, we suggest that a mere failure in mature egg production under starvation conditions [50] is related to anovulation more than to PCOS.

The triangular relationship captured in our Ob-T2D-OD model is of relevance to human PCOS. It is estimated that over 50% of women with PCOS are overweight or obese, and approximately 75% have impaired insulin sensitivity [24,25]. PCOS is a complex disorder involving both genetic factors and lifestyle factors, such as a high-caloric diet. Nutritional and metabolic state has a strong impact on reproduction and, in women with anovulatory PCOS, the defects in oocyte maturation and ovulation are significantly associated with T2D and IR. Our established Ob-T2D-OD model captures the pathophysiological responses to high caloric diet, thus resembling the metabolic subtype of PCOS. Metformin is regarded as a positive control of drug treatment in PCOS animal models [63,64], and it can partially rescue the egg-laying defect in our Ob-T2D-OD model (our unpublished results). This further supports a conservation between the mechanisms leading to the pathophysiology in our model and those in other animal models or humans.

Our Ob-T2D-OD model has enabled us to observe the temporal order of the three aspects of pathophysiology in a well-defined, quantitative manner. Our results suggest an early onset of Ob and that, while T2D may not be the primary cause for an oogenesis defect, it may promote its progression and further deterioration. These results point toward a complex nature of the interactions within the diet-induced triangular relationship. They further support the possibility of using a disease model in a genetically defined model organism to complement and guide human studies, particularly with respect to the heterogeneous nature of PCOS. For patients with the metabolic subtype of PCOS, lifestyle changes are considered the first-line therapy, including a reduction in calorie intake and exercise to reduce body fat [65]. It remains to be determined whether our Ob-T2D-OD model may also be of use in evaluating the “recovery” dynamics. In addition, genetic manipulations in *Drosophila* may be combined with the use or high-throughput screening [66] of chemicals toward disentangling the complex gene-environment interactions relevant to PCOS.

Recent studies support the utility of *Drosophila* models in investigating diet-induced pathophysiology ([8,15,43,44,67]; see also [68,69]). While previous studies have largely focused on the individual aspects of diet-induced pathophysiology, and our work takes a quantitative systems approach, focusing on all three aspects that constitute a clinically relevant triangular relationship, aiming specifically at OD and PCOS. Importantly, we have evaluated these aspects as integrated responses, as in the four distinct scenarios in our CCD modeling, and have systematically analyzed the onset timing of individual aspects in relation to each other. These led us to document not only the complex interactions between the two macronutrients—as well as the treatment duration—on impacting pathophysiology (see also [15,44]), but also a temporal order of the onset timing suggestive of an intercalating relationship in pathophysiological progression of OD. Our study, thus, further documents the power of quantitative systems approaches toward uncovering important new insights in biology.

## 4. Conclusions

The design of our current study has three underpinning features: (1) *a quantitative modeling approach*, which led to the identification of a diet treatment condition suitable for a specifically targeted disease model; (2) *a sharp focus* on a triangular relationship in *Drosophila*, which was aimed at understanding the biology of oogenesis in a well-controlled genetic system of relevance to human conditions, such as PCOS; and (3) *a dynamic framework*, which guided our investigation of the onset timing of three distinct—but interrelated—aspects of diet-induced pathophysiology. These features represent the core of a platform—into which other features, such as well-established genetic approaches in *Drosophila* may be incorporated—for mechanistic investigations in a model organism of clinical or pharmaceutical relevance.

## Data Availability

RNA sequencing data were deposited at SRA, under PRJNA866605.

## Supplementary Materials

The following supporting information can be downloaded at: https://www.mdpi.com/article/10.3390/nu14245365/s1. Figure S1: Statistical parameters for evaluating Bf and F; Table S1: Factors and levels of central composite design; Table S2: Central composite design with experimental values; Table S3. Analysis of variance in the model for Bf and F; Table S4: Continuous measurement of the average egg production; Table S5: Complete information of DEG, GO, and KEGG analyses.
