# Supplementary material for: Modeling Obesity-Associated Ovarian Dysfunction in Drosophila
Source: Nutrients. 2022 Dec 16;14(24):5365. doi: 10.3390/nu14245365 (PMC9783805; doi:10.3390/nu14245365)
Supplement: Supplementary file 1 [file nutrients-14-05365-s001.zip › nutrients-2079703-SI.pdf]

Supplementary Materials

Supplemental Figures

Fig.S1

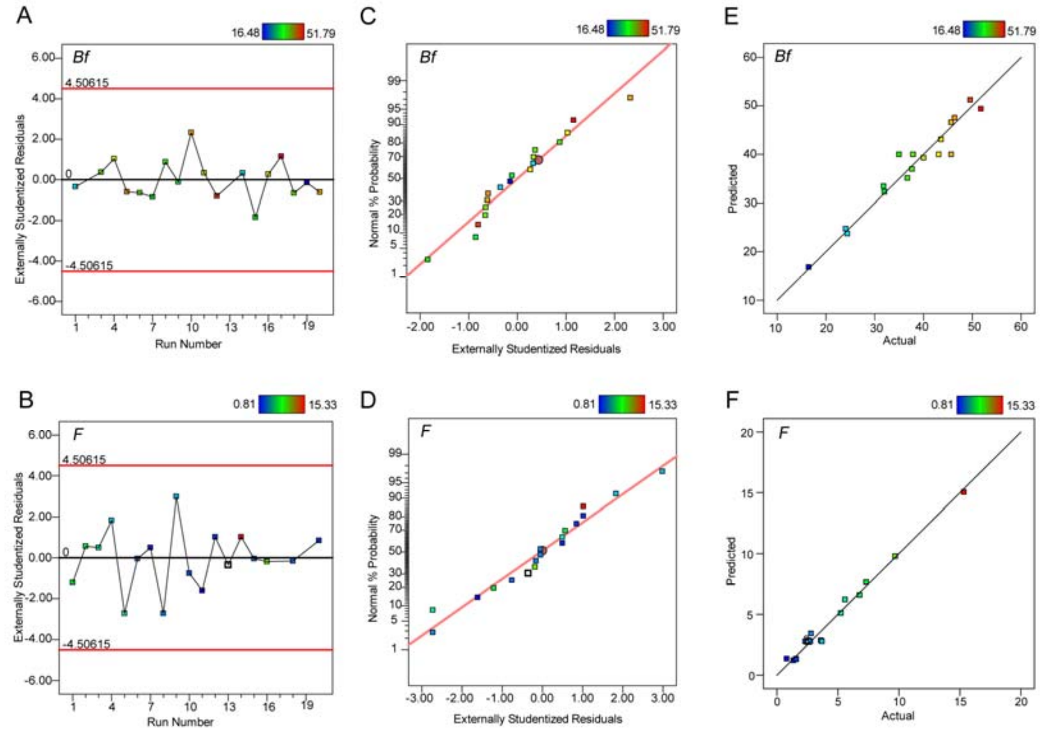

Figure S1. Statistical parameters for evaluating *Bf* and *F*

(A-B) Plots of the residuals versus the experimental run order for *Bf* (A) and *F* (B). The *X*-axis represents the experiment run number ranging from 1 to 20, and *Y*-axis represents the externally studentized residuals. Residuals from runs for model fitting showed no apparent trend. This indicates the lack of lurking variables that may influence the response.

(C-D) Normal Plot of Residuals for *Bf* (C) and *F* (D). The *X*-axis represents the externally studentized residuals, and *Y*-axis represents the normal probability. The graphs indicated approximately linear residual distributions.

(E-F) Plots of Predicted vs. Actual for *Bf* (E) and *F* (F). The *X*-axis represents the actual values tested, and *Y*-axis represents the predicted values tested. Dots show good linearity, representing the predicted values agreed the actual value.

Supplemental Tables

Table S1. Factors and levels of central composite design

Factor	Level				
	-α	-1	0	1	α
S: Sucrose (g/dL)	0.57	12.5	30	47.5	59.43
P: Yeast (g/dL)	0.32	1	2	3	3.68
D: Duration (d)	1.64	3	5	7	8.36

α= 1.682

Table S2. Central composite design with experimental values

Run	*S* (g/dL)	*P* (g/dL)	*D* (d)	*Bf* (nmol/mg)	*F* (n)
1	12.5	3	7	24.06±1.63	7.33±0.66
2	47.5	3	3	41.16±0.24	6.79±0.80
3	12.5	1	3	37.67±2.72	5.25±0.64
4	30	2	5	43.13±1.69	3.70±0.38
5	30	2	1.64	46.39±5.04	5.59±0.48
6	30	2	5	37.90±0.85	2.75±0.50
7	47.5	1	7	31.84±5.46	1.36±0.51
8	59.43	2	5	36.73±1.49	2.80±0.65
9	47.5	1	3	32.02±1.65	3.61±1.15
10	30	2	5	45.71±4.40	2.33±1.01
11	30	0.32	5	40.02±3.47	0.80±0.36
12	47.5	3	7	49.59±0.72	1.58±0.72
13	30	2	5	76.04±7.03	2.57±0.92
14	12.5	3	3	24.41±0.51	15.33±1.31
15	30	2	5	35.01±2.21	2.75±0.36
16	30	3.68	5	43.60±1.77	9.71±1.88
17	30	2	8.36	51.79±5.30	2.78±0.26
18	30	2	5	37.88±1.05	2.68±0.67
19	0.57	2	5	16.48±0.90	2.83±0.21
20	12.5	1	7	45.68±1.84	1.57±0.43

Table S3. Analysis of variance in the model for *Bf* and *F*

	Bf				*F*			
Source	Sum of Squares	Mean Square	F-value	p-value	Sum of Squires	Mean Square	F-value	p-value
**Model**	1406.64	156.29	13.26	0.0007	218.8625	24.3181	62.371	0.0000
x_1-sucrose	331.07	331.07	28.08	0.0007	40.2582	40.2582	103.2543	0.0000
x_2-yeast	14.46	14.46	1.23	0.3003	85.7943	85.7943	220.0453	0.0000
x_3-duration	3.39	3.39	0.2873	0.6065	44.721	44.721	114.7005	0.0000
x_1x_2	569.65	569.65	48.31	0.0001	19.3602	19.3602	49.6551	0.0001
x_1x_3	25.9	25.9	2.20	0.1766	2.2342	2.2342	5.7304	0.0436
x_2x_3	26.91	26.91	2.28	0.1693	6.6154	6.6154	16.9673	0.0033
$x_1{}^2$	325.19	325.19	27.58	0.0008	16.9719	16.9719	43.5295	0.0002
$x_2{}^2$	2.4	2.4	0.2036	0.6638	12.9333	12.9333	33.1713	0.0004
$x_3{}^2$	119.3	119.3	10.12	0.0130	0.1324	0.1324	0.3397	0.5761
**Residual**	94.33	11.79			3.1191	0.3899		
Lack of Fit	18.12	4.53	0.2738	0.9034	2.0252	0.6751	3.0854	0.1283
Pure Error	76.21	19.05			1.094	0.2188		
**Cor Total**	1500.96				221.9817			
**R^2-adjusted**	0.8665				0.9701			
**R^2-predicted**	0.7887				0.8511			
**Adeq Precision**	13.4419				29.7218			

Table S4. Continuous measurement of the average egg production

	Treatment_rep	0-1d	1-2d	2-3d	3-4d	4-5d	5-6d
Population_1 (3d mating)	S_2P_3_1	8.29	8.57	9.14	8.71	6.57	12.00
	S_2P_3_2	10.57	9.14	8.14	8.57	7.71	10.14
	S_2P_3_3	3.50	9.00	7.14	3.00	3.00	6.29
	$S_{35}P_3$_1	5.00	5.43	5.57	2.43	1.43	1.71
	$S_{35}P_3$_2	10.71	3.00	3.00	1.71	1.00	3.57
	$S_{35}P_3$_3	6.29	5.86	1.29	1.14	1.29	1.29
Population_2 (3d mating)	S_2P_3_4	10.86	4.00	6.00	7.57	7.86	3.86
	S_2P_3_5	11.71	8.43	4.43	6.00	5.29	5.57
	S_2P_3_6	13.86	9.00	12.00	11.86	6.86	7.57
	$S_{35}P_3$_4	10.57	5.29	3.14	1.57	1.86	0.57
	$S_{35}P_3$_5	9.71	2.00	1.43	2.86	2.86	1.29
	$S_{35}P_3$_6	8.86	1.29	2.86	1.29	1.29	0.86
